# Association between maxillary and mandibular apical base lengths and severity of dental crowding or spacing in Class II malocclusion subjects: An *in-vitro* study

**DOI:** 10.4317/jced.55422

**Published:** 2019-01-01

**Authors:** Rishi-Raj Singh, Pratik Verma, Devina Pradhan, Rishibha Bhardwaj, Simran Kour

**Affiliations:** 1 MDS. Consultant Orthodontist, Indiranagar, Lucknow, Uttar Pradesh, India; 2 MDS. Tutor, Department of Orthodontics & Dentofacial Orthopaedics, Dental Institute, Rajendra Institute of Medical Sciences, Ranchi, Jharkhand, India; 3 MDS. Senior Lecturer, Department of Public Health Dentistry, Rama Dental College Hospital & Research Centre, Kanpur, Uttar Pradesh, India; 4 MDS. Senior Lecturer, Department of Orthodontics & Dentofacial Orthopaedics ITS Dental College, Ghaziabad, Uttar Pradesh, India; 5 MDS. Consultant Orthodontist, Sanjay chowk Jammu, India

## Abstract

**Background:**

Anterior crowding/ spacing are one of the major problems that inspire patients to undergo orthodontic treatment. Several factors associated with anterior crowding/ spacing includes arch width and length, mesio-distal tooth diameter and proportions. The aim of the present study was to evaluate the relationship of maxillary and mandibular base lengths to the amount of anterior dental crowding/ spacing in patients with complete class II malocclusions.

**Material and Methods:**

A retrospective study was done with 152 patients who were divided into four groups according to the severity of pre-treatment dental crowding/spacing present in the mandibular arch. Measurements were performed on the pre-treatment dental casts and lateral cephalograms. Statistical analysis was done using t-test and chi-square test.

**Results:**

Subjects with complete class II malocclusion and moderate to severe mandibular crowding [≥3mm] have significantly smaller base lengths as compared to the subjects with slight crowding or spacing [<3mm].

**Conclusions:**

There exists an inverse correlation between maxillary and mandibular base lengths and the severity of dental crowding and direct correlation with spacing.

** Key words:**Apical base lengths, Class II malocclusion, Crowding, Incisor inclination, Spacing.

## Introduction

Anterior crowding is one of the most common problems that motivate patients to seek orthodontic treatment. Many factors have been evaluated and found to be related to anterior dental crowding, including dental arch width and length, mesio-distal tooth diameter and dental proportions (1). However, incisor crowding is not merely a tooth-arch size discrepancy. Several other variables such as direction of mandibular growth, early loss of deciduous molars, the oral and peri-oral musculature, incisor and molar inclination can be associated with crowding (2).

The association between dental crowding and tooth size has been studied previously, however conclusions remain discrepant. It is expected that tooth size is not the only determining factor in the origin of dental crowding (3). Laskin and Schulhof have suggested that lower anterior crowding is strongly associated with eruption of lower third molars (4). In an investigation performed by Howe *et al.*, comparisons made between crowded and non-crowded groups using study models indicated that arch dimensions made a greater contribution to dental crowding than tooth size (5).

Crowding is often related to arch dimensions. Only few studies evaluated the relationship between crowding and cephalometric measurements. Sakuda *et al.* reported a significant correlation between an increase in lower incisor crowding and high mandibular plane angles, short mandibular body lengths, great upper face height and small vertical dimensions in the upper posterior segments (2).

Incisor inclination is yet another factor that was considered while determining the causes of spacing/crowding in dental arch. Sanin and Savara, in a study of factors affecting mandibular incisor alignment noted that a vertical or lingual inclination of lower incisors in the mixed dentition is associated with crowded incisors in the permanent dentition. In yet another study conducted by Lundstrom, where he examined twenty-five pairs of twins to study the changes in incisor inclination between 12 to 15 years and 23 to 26 years, it was found that the age changes for spacing/crowding were correlated with neither change in incisor inclination nor growth direction of the mandible (1,2).

In general, patients with class II malocclusion have a smaller mandibular length than subjects with normal occlusion and class I malocclusion. However, the relationship between apical base length and anterior dental crowding in a sample class II malocclusion exclusively has not been investigated.(2) Considering the above background, the aim of the present study is to evaluate the relationship of maxillary and mandibular base lengths to the amount of anterior dental crowding/ spacing in patients with complete class II malocclusions.

## Material and Methods

-Sample selection 

 The present study was conducted in the postgraduate department of Orthodontics and Dentofacial Orthopaedics at Rama Dental College, Hospital and Research Centre, Kanpur, India. The sample was retrospectively selected from the orthodontic department of Rama Dental College, Hospital and Research Centre, Kanpur.

-Approval from authorities

The study protocol was approved by the Institutional Review Board of Rama Dental College, Hospital and Research Centre, Kanpur. A written informed consent was taken from all the patients who participated in the study.

The inclusion criteria were as follows.

1. Presence of a complete (full cusp) bilateral class II malocclusion molar relationship

2. Presence of all permanent teeth up to the first molars

The exclusion criteria were as follows.

1. No open bite or cross bite 

2. Absence of proximal decay or restoration

3. Absence of dental anomalies of number, size, form and position

4. Absence of any disease or syndrome with dental manifestations

5. Absence of clefts of lip or palate

-Study design

152 patients who satisfied the inclusion criteria were selected. The sample was then divided into four groups according to the severity of pre-treatment dental crowding/spacing present in the mandibular arch.

Group 1 - 40 patients (20 males and 20 females) with crowding < 3mm.

Group 2 - 39 patients (21 males and 18 females) with crowding ≥ 3mm.

Group 3 - 38 patients (22 males and 16 females) with spacing < 3mm.

Group 4 - 35 patients (15 males and 20 females) with spacing ≥ 3mm.

-Recording of measurements

Measurements were performed on the pre-treatment dental casts and lateral cephalograms. The cephalograms of the subjects were taken using Orthorelix 9200 (Gendex) cephalometric cum orthopantomograph machine. All lateral cephalograms were taken on the same radiographic unit and were traced by single operator. Tracing was done on acetate sheets using RotringTM 0.35” pencil. Condylion (Co), Point A, Gnathion (Gn) were some of the cephalometric landmarks that were used in the study. The linear measurements used were Co-Point A and Co-Gn. Standard orthodontic impression trays were used for impression taking.

Mandibular and maxillary crowding/spacing were calculated as the difference between arch perimeter and the sum of tooth widths from second premolar to second premolar on the other side. The values were calculated in millimetres and were calculated by a single examiner. The arch perimeter was equal to the sum of tooth widths in a well aligned arch. Negative values indicated crowding, whereas values with arch perimeter being greater than the sum of tooth widths indicated spacing.

A set of maxillary and mandibular dental casts from each patient was taken. A vernier calliper calibrated with digital micrometer was used to measure the mesio-distal widths of individual teeth. In addition, the arch perimeter for maxillary and mandibular dental arches was measured using a brass wire and a set of maxillary and mandibular pre-treatment dental casts.

-Statistical Analyses

Intergroup compatibility for initial age and sex distribution was evaluated with t-tests and chi-square tests respectively. The cephalometric variables were compared between the groups with t-tests. Correlation between the maxillary and mandibular effective lengths and dental crowding and spacing severity was investigated with the Pearson correlation coefficient. Significance level was fixed at *p* < 0.05.

-Errors in the study

A month after the first measurements were taken, 80 pairs of dental casts (20 of each group) were taken and re-measured. 80 randomly selected lateral cephalograms were retraced by the same examiner. Casual errors were calculated according to Dahlberg’s formula.

## Results

Subjects with moderate to severe dental crowding (≥3mm) showed smaller maxillary and mandibular apical base lengths (81.41 mm and 104.28 mm respectively) as compared to those with mild (<3mm) or no dental crowding ( Table 1).

**Table 1:**
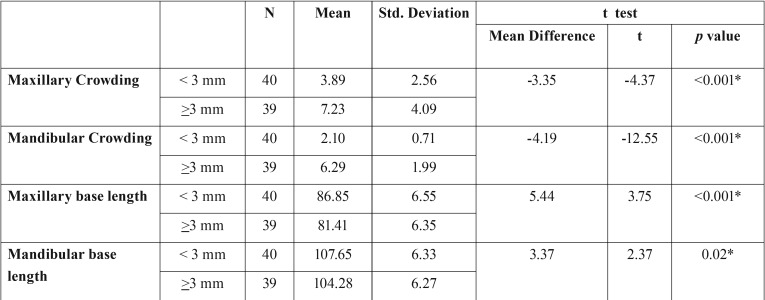
Subjects with moderate to severe dental crowding (≥3mm) had smaller maxillary and mandibular base lengths (81.41 mm and 104.28 mm respectively) as compared to those with mild (<3mm) or no dental crowding.

A weak to moderate inverse correlation was found between the amount of dental crowding and maxillary and mandibular base lengths. An inverse correlation of -0.377 was observed between mandibular crowding and maxillary base length (Co-Pt A), and -0.247 between mandibular crowding and mandibular base length (Co-Gn). Strong positive correlations of 0.570 and 0.608 were found between maxillary and mandibular crowding and between maxillary and mandibular base lengths respectively.

In groups with dental spacing, it was seen that the subjects with moderate to severe (≥3mm) dental spacing have larger maxillary and mandibular apical base lengths (98.53 mm and 118.89 mm respectively), as compared to those with slightly (<3mm) spaced dentition i.e. 92.50 mm for maxillary and 113.41 mm for mandibular base lengths. A positive correlation factor of 0.266 and 0.324 were observed between mandibular spacing and maxillary and mandibular base lengths respectively ( Table 2, Table 3).

**Table 2:**
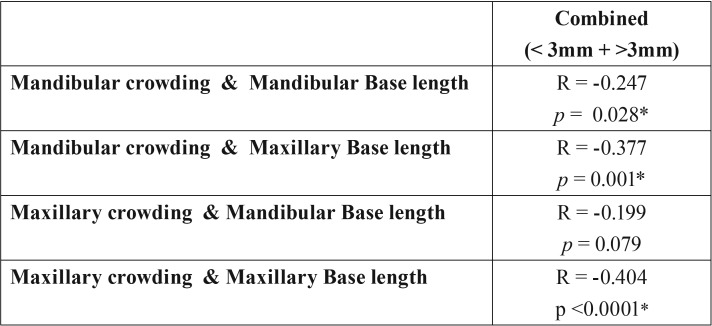
Inverse correlation between crowding and base lengths.

**Table 3:**
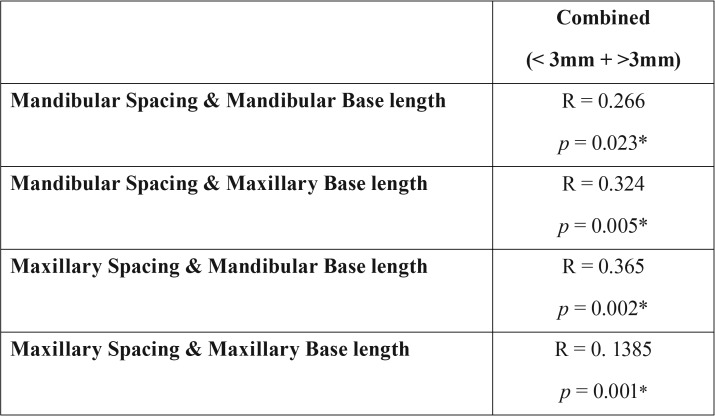
Direct correlation between spacing and base lengths.

## Discussion

Orthodontic diagnosis relies not only upon anatomic relations, physiologic and biologic factors, but also upon aesthetic judgement. Visualising orthodontic diagnosis as an exact science and predicating it upon such variables will be like casting all the individuals in a common mould (6,7). An assessment of the amount of crowding or spacing of anterior teeth is one of the first steps in orthodontic diagnosis and treatment planning. Objective information on the amount of arch size-tooth-size discrepancy in the incisor region often tips the balance for or against premolar extraction (8). Leighton BC and Hunter and Keeling *et al.* reported on the relationship between dental crowding and the morphological characteristics of the mandible. They observed a smaller mandibular body length in patients with severe crowding in the mixed and permanent dentition (6,9).

A weak to moderate inverse correlation was found between the amount of dental crowding and maxillary and mandibular base lengths. Strong positive correlations of 0.570 and 0.608 were found between maxillary and mandibular crowding and between maxillary and mandibular base lengths respectively. These results are similar to the results seen in a previous study conducted by Janson *et al.* (2011) (1). Similar results were also seen in some previous studies, where the malocclusion was not specified.

In case of the groups with dental spacing, it was seen that the subjects with moderate to severe (≥3mm) dental spacing have larger maxillary and mandibular apical base lengths (98.53 mm and 118.89 mm respectively), as compared to those with slightly (<3mm) spaced dentition i.e. 92.50 mm for maxillary and 113.41 mm for mandibular base lengths. A positive correlation factor of 0.266 and 0.324 were observed between mandibular spacing and maxillary and mandibular base lengths respectively. Even stronger correlations were observed between maxillary spacing and the apical base lengths. A correlation of 0.385 was observed between maxillary spacing and maxillary base length, whereas a correlation factor of 0.365 was observed between maxillary spacing and the mandibular apical base length. Hemley (1971), however, considered the presence of dental spacing in one third of the population as a normal variation. In an epidemiologic study conducted by Steigman and Weissberg (1985) they found that 48.6% of individuals, out of a total sample size of 1279 subjects had at least one interdental space present, thus supporting Hemley’s claim of considering spaced dentition as a variation of the normal occlusion (10).

A serial study of changes in spacing between teeth during the period of facial maturation by Steigman, Gershkovitz and Harari (1985) concluded that 79% of the antecedent spaced dentitions in young adolescents remained through the maturation of occlusion. However, the distribution of spaces between various teeth proved to be unstable, at least during the period of occlusal maturation. Some spaces closed while others opened up. This finding provided an indication towards mesial or distal migration of teeth (11). While the spaces distal to the canine tended to close, new spaces mesial to the tooth opened up. Bishara and Andreasen (1989) attributed it to the eruption of the third molars and their tendency to migrate mesially, whereas Ng and Picton rendered the tendency of cuspids and bicuspids to migrate distally, as the cause for this behaviour. They found maxillary midline diastema to be the most stable space which showed no probability for spontaneous closure when it persisted after the full eruption of the cuspids (12).

As stated by Richardson, Adams, and McCartney, the discrepancies in measuring an apparently simple entity, such as the width of a tooth, may be surprisingly large. In their study they found that a single operator could reproduce his measurements with an acceptable degree of accuracy, whereas the discrepancies between measurements made by different operators were larger (13-16). Thus, a single operator made the measurements in the current study. The error variance for the current study is 5%, which is acceptable, and was calculated using Dahlberg’s formula.

It was, however, a matter of imperative curiosity to establish a relationship of apical bases of maxilla and mandible to different degrees of dental crowding as well as spacing amongst patients having class II malocclusion. The present study was thus conducted with keeping the foresaid goal in mind.

The accurate evaluation of error of measurement (EM) is extremely important in growth studies as well as clinical research, since there are usually quantitatively small changes. In any study it is important to evaluate the EM to validate the results and, consequently, the conclusions. Because of its extreme simplicity, the Dahlberg formula is largely used worldwide, mainly in cephalometric studies. In dentistry, in order to interpret the results of a study, the author has to consider how imprecise it is to trace landmarks. In both studies of growth and in clinical trials, the changes are subtle, which makes the error of the method quite important. In order to evaluate the variance of error between researches, several authors suggested the formula proposed by Dahlberg in 1940.

Midtgård J and others stated that the error variance should not exceed 3% of the total and if it exceeds 10%, then the method of measurement was inappropriate. However, Houston in his study of analysis of errors in orthodontic measurements, stated that this was an idealistic goal and cannot always be achieved (17,18).

Based on the findings of the current study, another inference may also be fashioned that, the shorter the apical base length, the greater the likelihood for crowding, and the larger the apical base length, the greater the likelihood for spacing. This is especially applicable to individuals with complete class II malocclusion, but it can be extrapolated to other types of malocclusions based on similar studies (1,2,6,19,20).

Although the correlation between dental crowding and apical base length is supported by some studies of the past not much work could be found in relation to dental spacing and base lengths, especially for class II malocclusion (19,20). But, since the results of one parameter (crowding) of the present study correlates well with other studies it may be presumed that the relation between dental spacing and effective base length established in this study holds true for other populations too, as it does for North Indian population (2,6,19,20).

It is noteworthy that although the groups were selected based on mandibular crowding and spacing, maxillary crowding and spacing were also significantly greater in the severely crowded and severely spaced groups respectively. This seems reasonable because in both the cases (crowding and spacing), there was a significant positive correlation between maxillary and mandibular effective base lengths and maxillary and mandibular crowding/spacing.

## Conclusions

The results of the present study reveal that the subjects with complete class II malocclusion and moderate to severe mandibular crowding have significantly smaller base lengths as compared to the subjects with same malocclusion with slight crowding or spacing.

Strong positive correlations were found between maxillary and mandibular crowding and between maxillary and mandibular base lengths respectively. Subjects with complete class II malocclusion and moderate to severe mandibular spacing have significantly larger base lengths as compared to the subjects with same malocclusion with slight spacing or mandibular crowding.

## References

[1] Janson G, Goizueta OEFM, Garib DG, Janson M (2011). Relationship between maxillary and mandibular base lengths and dental crowding in patients with complete class II malocclusions. Angle Orthod.

[2] Turkkahraman H, Sayin MO (2004). Relationship between mandibular anterior crowding and lateral dentofacial morphology in the early mixed dentition. Angle Orthod.

[3] Bernabe E, Flores-Mir C (2006). Dental morphology and crowding. A multivariate approach. Angle Orthod.

[4] Kavra TR, Kabra E (2013). A clinical and cephalometric study of the influence of mandibular third molars on mandibular anterior teeth. J Ind Orthod Soc.

[5] Poosti M, Jalali T (2007). Tooth size and arch dimension in uncrowded versus crowded class I malocclusions. J Contemp Dent Pract.

[6] Leighton BC, Hunter WS (1982). Relationship between lower arch spacing/crowding and facial height and depth. Am J Orthod.

[7] Carey CW, Alto P, Calif (1951). Diagnosis and case analysis in orthodontics. Am J Orthod.

[8] Harris EF, Vaden JL, Williams RA (1987). Lower incisor space analysis: A contrast of methods. Am J Orthod Dentofacial Orthop.

[9] Shigenobu N, Hisano M, Shima S, Matsubara N, Soma K (2007). Patterns of dental crowding in the lower arch and contributing factors. Angle Orthod.

[10] Steigman S, Weissberg Y (1985). Spaced Dentition: An epidemiologic study. Angle Orthod.

[11] Steigman S, Gershkovitz E, Harari D (1985). Characteristics and stability of spaced dentition. Angle Orthod.

[12] Bishara SE, Jakobsen JR, Treder JE, Stasi MJ (1989). Changes in the maxillary and mandibular tooth size-arch length relationship from early adolescence to early adulthood. A longitudinal study. Am J Orthod Dentofacial Orthop.

[13] Richardson A (1966). An investigation into the reproducibility of some points, planes and lines used in cephalometrics analysis. Am J Orthod.

[14] Richardson ME (1994). The etiology of late lower arch crowding alternative to mesially directed forces: a review. Am J Orthod Dentofacial Orthop.

[15] Richardson ME (1995). Late lower arch crowding: the role of the transverse dimension. Am J Orthod Dentofacial Orthop.

[16] Richardson ME (1999). A review of changes in lower arch alignment from seven to fifty years. Semin Orthod.

[17] Midtgård J, Björk G, Linder-Aronson S (1974). Reproducibility of cephalometric landmarks and errors of measurements of cephalometric cranial distances. Angle Orthod.

[18] Houston WJ (1983). The analysis of errors in orthodontic measurements. Am J Orthod.

[19] Sakuda M, Kuroda Y, Wada K, Matsumoto M (1976). Changes in crowding of the teeth during adolescence and their relation to growth of the facial skeleton. Trans Eur Orthod Soc.

[20] Berg R (1986). Crowding of the dental arches: A longitudinal study of the age period between 6 and 12 years. Eur J Orthod.

